# Endothelial nitric oxide synthase levels and their response to exercise in patients with slow coronary flow

**DOI:** 10.5830/CVJA-2013-072

**Published:** 2013-11

**Authors:** Hakan Taşolar, Erdal Aktürk, Ferhat Eyyüpkoca, Mehmet Cansel, Jülide Yağmur, Hasan Pekdemir, Yasin Karakuş, Fatma Özyalin, Burak Altun

**Affiliations:** Department of Cardiology, Adiyaman University, Training and Research Hospital, Adiyaman, Turkey; Department of Cardiology, Adiyaman University, Training and Research Hospital, Adiyaman, Turkey; Department of Cardiology, Faculty of Medicine, Inonu University, Malatya, Turkey; Department of Cardiology, Faculty of Medicine, Inonu University, Malatya, Turkey; Department of Cardiology, Faculty of Medicine, Inonu University, Malatya, Turkey; Department of Cardiology, Faculty of Medicine, Inonu University, Malatya, Turkey; Malatya State Hospital, Malatya, Turkey; Department of Medical Biochemistry, Faculty of Medicine, Inonu University, Malatya, Turkey; Department of Cardiology, Faculty of Medicine, Mart University, Canakkale, Turkey

**Keywords:** endothelial nitric oxide synthase, slow coronary flow, endothelial dysfunction, exercise, rate–pressure product

## Abstract

**Background:**

Endothelial dysfunction plays a key role in the aetiopathogenesis of slow coronary flow (SCF) even if there is no obstructive epicardial lesion. Reduced plasma levels of endothelial nitric oxide synthase (eNOS) are an important indicator of endothelial dysfunction. We aimed to determine plasma levels of eNOS and their relationship with exercise in patients with SCF.

**Methods:**

Twenty-two patients with SCF in at least one coronary artery and 17 healthy individuals were included in this study. The TIMI frame count method was used to determine SCF. Plasma levels of eNOS before and after effort were determined in the patient and control groups.

**Results:**

Basal eNOS levels in the patient group were lower than in the control group (*p* = 0.040), and plasma eNOS levels after exercise decreased more significantly in the patient group compared to the control group (*p* = 0.002). Median decreases of eNOS in response to exercise were higher in the SCF group than in the control group (*p* < 0.001), and the decrease observed in the control group was not statistically significant (*p* = 0.35). There were significantly negative correlations between TIMI frame count and plasma levels of eNOS at baseline and after exercise (*r* = –0.51, *p* = 0.015, *r* = –0.58, *p* = 0.005, respectively). Moreover, there was also a positive correlation between the rate–pressure product and plasma levels of eNOS after exercise in patients with SCF (*r* = 0.494, *p* = 0.019).

**Conclusion:**

Our findings indicate an important pathophysiological relationship between the severity of SCF in which endothelial dysfunction plays a role in its pathogenesis and the level of circulating plasma levels of eNOS.

## Abstract

Slow coronary flow (SCF), described for the first time by Tambe and his colleagues in 1972, is an angiographic diagnosis characterised by a low rate of flow of contrast agent in the epicardial coronary arteries, together with typical angina pectoris and normal coronary arteries.^1^ Even though micro- and macrovascular disease findings have been identified, such as myofibrillar hypertrophy, myofibrillar degeneration, hyperplastic fibromuscular thickening, luminal narrowing, endothelial degeneration, endothelial dysfunction and diffuse atherosclerosis, which may lead to reduced coronary flow reserve, uncertainties still exist in the aetiopathogenesis.^2^,^3^

Coronary blood flow and oxygen transport to the myocardium are increased by autoregulatory mechanisms for the increased metabolic needs associated with effort. The amount of oxygen extracted from the blood also increases, which leads to a decrease in the concentration of oxygen in the blood. Mitochondrial metabolism is altered by coronary endothelium-derived nitric oxide (NO) in an attempt to reduce the growing energy requirements.^4^,^5^

Vascular endothelium exhibits a number of haemostatic functions in normal blood vessels. NO is a key molecule for normal autoregulatory mechanisms, such as modulating the vasodilator response to tachycardia and exercise,^6^ and it has also been found to be essential for flow-mediated dilatation of large human arteries *in vivo*.^7^ Endothelial nitric oxide synthase (eNOS) is an enzyme involved in the synthesis of NO.^8^ Decreased plasma eNOS level is an important indicator of endothelial dysfunction.^9^

To our knowledge, there has been no study evaluating plasma eNOS levels and their response to exercise in SCF patients. Therefore we aimed to investigate the plasma levels of eNOS before and after exercise in patients with SCF.

## Methods

Twenty-two patients (19 men, three women, mean age 48.5 ± 10.9 years) with angiographically proven SCF in at least one coronary artery but normal epicardial coronary arteries were enrolled in this study. Seventeen age- and gender-matched patients (12 men, five women, mean age 48.7 ± 9.6 years) who had undergone coronary angiography because of typical and quasi-typical symptoms of angina, with normal coronary arteries and normal coronary flow on coronary angiography comprised the control group. All control subjects had no history of cardiovascular disease, and normal echocardiographic and exercise studies. Because diet affects plasma eNOS levels,^10^,^11^ none of the participants were provided any dietary programme.

The study was conducted according to the guidelines of the Declaration of Helsinki and was approved by the Ethics Research Committee of Inonu University Faculty of Medicine. Informed consents were obtained from all the participants.

Exclusion criteria for the study were defined as the presence of occlusive coronary artery disease in at least one coronary artery, valvular heart disease, blood pressure above 140/90 mmHg, cardiac arrhythmia, atrio-ventricular conduction abnormalities, congestive heart failure or cardiomyopathy, usage of any medication (e.g. statins, aspirin, beta-blockers, digoxine, non-steroidal anti-inflammatory drugs, warfarin, antidepressant medication, corticosteroids, insulin, oral antidiabetic drugs), chronic liver and renal disease, obesity, diabetes mellitus, chronic obstructive pulmonary disease, peripheral artery disease, congenital heart disease and an additional systemic disease.

A standard coronary angiography procedure was performed on all participants through the femoral and radial artery with a Philips Integris 5000, Netherlands, coronary angiography device. Results of the coronary angiography were assessed by two blinded observers unaware of the patients’ plasma eNOS levels.

The TIMI frame count method was used for the detection of SCF and measurement of opaque material.^12^ The time required for contrast to reach the distal decisive points of a coronary artery was expressed as frame count. The starting point was the moment when the contrast agent began to move forward contacting both sides of the artery. The end points were: for the left anterior descending artery (LAD), when the contrast agent had reached the branch point of the artery, called the mustache; for the right coronary artery (RCA), the point where the posterolateral artery has its first side branch; and for the circumflex artery (Cx), the point where the longest branch has a distal bifurcation.

Because the LAD has a longer course than the other arteries, the calculated value was standardised by dividing it by 1.7. SCF was defined as the patients having frame count values above the standard deviations for at least one coronary artery: 36.2 ± 2.6 for the LAD, 22.2 ± 4.1 and for the Cx, and 20.4 ± 3.0 for the RCA.^12^

Sub-maximal exercise stress tests (EST) using the Bruce protocol (200 or until the maximal heart rate minus 15%) were performed on both groups after coronary angiography was completed. All medications taken by the patient and control groups were stopped for at least five half-lives before the test.

Strength applied during the EST protocol was automatically calculated via an installed computer program according to the formula: maximal heart rate = 220 – age (years), depending on the participant’s maximal heart rate. Achievement of maximal heart rate, declaring of intolerable workload during the test and formation of any clinical indications (e.g. onset of typical chest pain, ≥ 0.1 mV horizontal or down-sloping ST-segment depression) were considered reasons for termination of the EST.

Blood pressure was measured every other minute with a manual sphygmomanometer (Erka series, Dusseldorf, Germany). All readings for blood pressure and heart rate were taken by experienced technicians. Total cholesterol, high-density lipoprotein and low-density lipoprotein cholesterol, triglyceride, leukocyte, glucose, blood urea nitrogen and creatinine values of the patient and control samples were measured with biochemical analyses.

Blood samples were obtained at rest and one minute after the exercise testing, using a 19-gauge needle by direct venipuncture, and drawn into 10-ml vacutainer tubes at room temperature containing K3-EDTA at rest and one minute after exercise testing. Sampling time was determined according to the study by Foote *et al.*^13^ The vacutainer tube was filled to capacity and gently inverted five times to ensure complete mixing of the anticoagulant. Then the sample was centrifuged at 1 000 rpm for 15 minutes. The resulting platelet-poor plasma was collected in 1.5-ml Eppendorf tubes and frozen at –40°C for biomarker assays.

All samples were drawn and analysed by blinded technicians on the day of the study. After collecting all the samples, plasma levels of eNOS were determined using a commercially available sandwich enzyme immunoassay kit (Uscn Life Science Inc, Wuhan, China, E90868Hu, L101129537). The minimum detectable dose of NOS_3_ for this assay is less than 5.5 pg/ml. The measurable range of the eNOS assay was 15.6 to 1 000 pg/ml. Each sample was measured in duplicate, and the overall intraassay coefficient of variation was calculated. The intra-assay coefficients of variation were 3.6%.

## Statistical analysis

Data analyses were performed using SPSS statistical software version 17.0 (SPSS Inc., Chicago, IL, USA). Variable values were expressed as ± standard deviation and categorical values were expressed as percentage. Categorical variables between the two groups were compared by chi-square test and continuous variables were compared by independent Student’s *t*-test. Paired *t*-test was used for comparison of plasma eNOS levels and exercise parameters and their response to exercise in the study population. Correlations of continuous variables were evaluated using Pearson’s correlation test. A *p*-value < 0.05 was considered significant.

## Results

Clinical and demographic characteristics of patient and control groups are given in Table 1. There was no significant difference between the groups in terms of mean age, gender, systolic and diastolic blood pressure, total cholesterol levels, smoking, family history, and coronary artery disease history.

**Table 1:** Clinical Characteristics Of Patient And Control Groups

*Clinical characteristics*	*SCF (n = 22)*	*Control (n = 17)*	p*-value*
Age (year)	48.5 ± 10.9	48.7 ± 9.6	NS
Male gender, *n* (%)	19 (86.4)	12 (70.6)	NS
BMI (kg/m^2^)	23.4 ± 1.7	23.2 ± 1.6	NS
Systolic blood pressure (mmHg)	116.5 ± 7.2	117.0 ± 6.8	NS
Diastolic blood pressure (mmHg)	76.2 ± 4.9	73.4 ± 5.2	NS
Smoking, *n* (%)	14 (63.6)	11 (58.8)	NS
Family history, *n* (%)	13 (54.2)	9 (60.0)	NS
Fasting blood glucose (mg/dl)	87.0 ± 7.5	87.3 ± 9.2	NS
Total cholesterol (mg/dl)	174.1 ± 24.1	180.6 ± 30.0	NS
LDL cholesterol (mg/dl)	117.8 ± 36.8	121.0 ± 31.7	NS
HDL cholesterol (mg/dl)	40.8 ± 8.4	36.3 ± 8.0	NS
Triglyceride (mg/dl)	99.0 ± 27.6	111.5 ± 31.5	NS
Ejection fraction (%)	61.1 ± 3.8	61.1 ± 3.9	NS
TIMI frame count	49.4 ± 11.7	19.9 ± 5.9	< 0.001

SCF: slow coronary flow, LDL: low-density lipoprotein, HDL: high-density lipoprotein, TIMI: thrombolysis in myocardial infarction, NS: not significant.

Because of chest pain and more than 2-mm ST-segment depression, the EST was terminated in seven SCF patients. Three had both chest pain and ST-segment depression, and four had only chest pain. During the EST as well as during the angiographic process, no chest pain was experienced in the control group. Baseline heart rate, peak exercise heart rate, peak exercise systolic blood pressure and rate–pressure product at baseline and after exercise were evaluated in both groups and are given in Table 2.

**Table 2:** Exercise Parameters Of Study Population

*Exercise parameters*	*Controls (n = 17)*	*SCF (n = 22)*	p*-value*
Baseline heart rate (bpm)	70.1 ± 2.8	69.0 ± 3.6	NS
Peak exercise heart rate (bpm)	185.9 ± 10.8	159.5 ± 10.3	< 0.001
Peak systolic blood pressure (mmHg)	193.6 ± 10.8	179.9 ± 9.3	< 0.001
Baseline rate–pressure product	81.6 ± 5.6	80.6 ± 5.3	NS
Peak exercise rate–pressure product	360.3 ± 32.4	287.2 ± 26.5	< 0.001
Angina, *n* (%)	–	4 (18)	–
ST segment depression, *n* (%)	–	7 (32)	–
Both angina and ST depression, *n* (%)	–	3 (14)	–

SCF: slow coronary flow, NS: not significant.

**Fig. 1.**
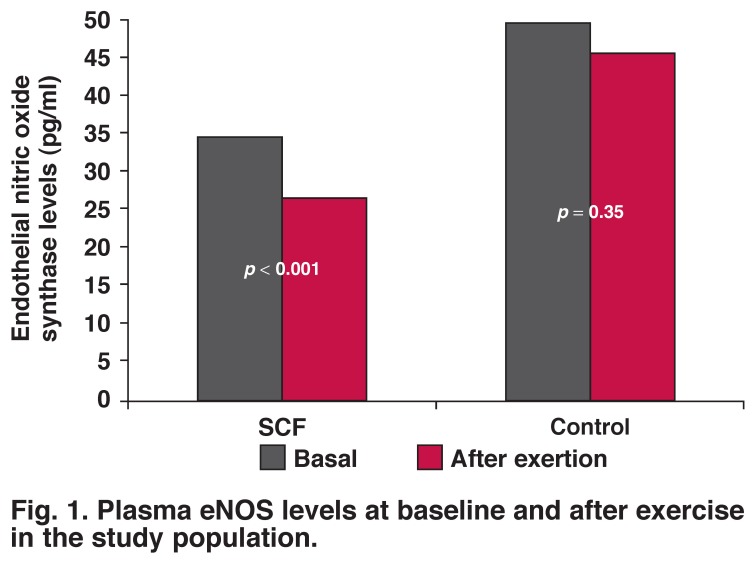
Plasma eNOS levels at baseline and after exercise in the study population.

Baseline and post-exercise plasma levels of eNOS in the patient and control groups are given in Table 3. Basal eNOS levels in the patient group were lower than in the control group (*p* = 0.040), and plasma eNOS levels after exercise were more significantly decreased in the patient group compared to the control group (*p* = 0.002). Median decreases in eNOS level in response to exercise were higher in the SCF group than in the control group (*p* < 0.001), and the decrease observed in the control group was not statistically significant (*p* = 0.35) (Fig. 1).

**Table 3:** Basal And Post-Exertion Plasma BNP, CRP And ENOS Levels

eNOS (pg/ml)*	*SCF (n = 22)*	*Control (n = 17)*	p*-value*
Basal	32.58 ± 21.36	48.16 ± 24.35	0.040
Post-exertion	25.02 ± 17.69	44.13 ± 17.39	0.002

SCF: slow coronary flow, eNOS: endothelial nitric oxide synthase, NS: not significant. *p < 0.001 (baseline and after exercise in patients with SCF).

**Fig. 2.**
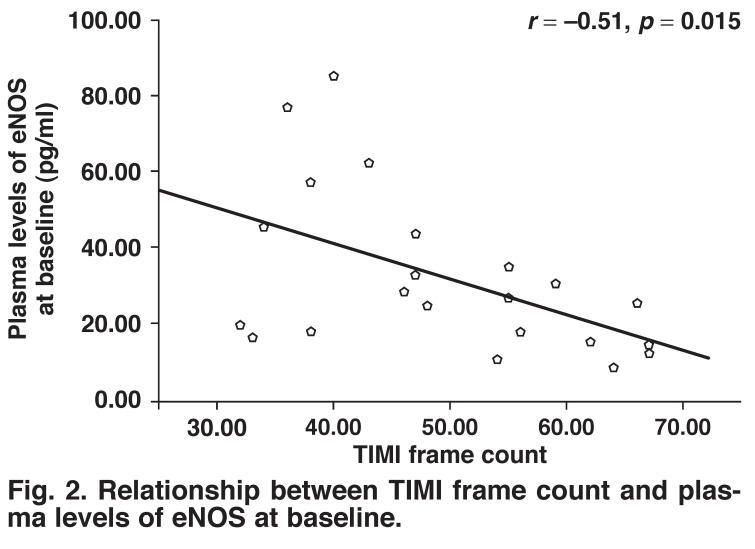
Relationship between TIMI frame count and plasma levels of eNOS at baseline.

**Fig. 3.**
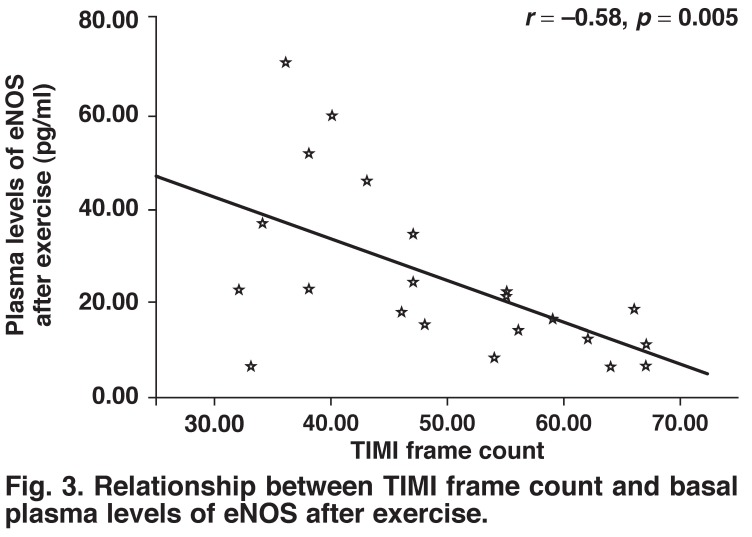
Relationship between TIMI frame count and basal plasma levels of eNOS after exercise.

**Fig. 4.**
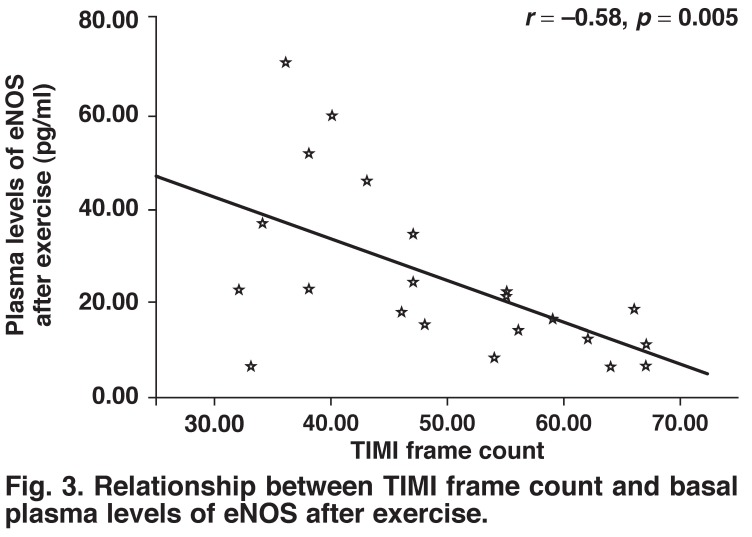
Relationship between rate–pressure product and plasma levels of eNOS.

There were significantly negative correlations between the TIMI frame count and plasma levels of eNOS at baseline and after exercise (*r* = –0.51, *p* = 0.015, *r* = –0.58, *p* = 0.005, respectively) (Figs 2, 3). Moreover, there was a positive correlation between the rate–pressure product and plasma levels of eNOS after exercise in patients with SCF (*r* = 0.494, *p* = 0.019) (Fig. 4).

## Discussion

The main objective of our study was to assess plasma eNOS levels and their response to exercise in patients with SCF. The main findings of our study were: (1) plasma levels of eNOS were lower in SCF patients than in control subjects, (2) the differences in eNOS levels between the two groups became greater after the exercise treadmill test, as a result of a significant decrease in plasma eNOS levels in patients with SCF, (3) there were significantly negative correlations between TIMI frame count and plasma levels of eNOS at baseline and after exercise, and (4) there was a positive correlation between the rate–pressure product and plasma levels of eNOS after exercise in SCF patients.

SCF is a pathology that causes typical angina pectoris and decreases the flow velocity of contrast agents in the coronary arteries of patients with normal coronary angiography.1 Despite well-defined angiographic characteristics of SCF, knowledge on its clinical significance and aetiopathogenesis are insufficient.

Occlusive disease of the small vessels, microvascular and/or endothelial dysfunction have been over-emphasised in the aetiology of this disease. It was shown in previous studies that the pathophysiology of SCF was at the microvascular level and the disease has a dynamic character.^1^, ^2^,^14^ A microcirculatory disorder of SCF was also clearly demonstrated in the results of these studies.^3^,^15^

The effects of exercise on the coronary microvascular tone are controversial. Sympathetic activation increases coronary flow, with both increasing heart rate and myocardial contractility,^16^ and endothelium-mediated vasodilation.^17^ Besides, increased sympathetic stimulation may also cause abnormal microvascular constriction in endothelial dysfunction.^18^ Essentially, the net effect of exercise is related to the pathophysiological state of the small coronary arteries.

The endothelium normally displays a vasodilatory feature against various systemic, neurohumoral and mechanical stimuli, and regulates vasomotor tension, thrombosis, fibrinolysis, vascular cell growth, and leukocyte and platelet adhesion by secreting growth factors and inhibitors such as NO.^19^ NO is a key molecule in normal autoregulatory mechanisms such as modulating the vasodilator response to tachycardia and exercise.^6^ NO formed via eNOS plays a crucial role in the regulation of coronary blood flow, resulting in reduction of vascular resistance by vasodilation and in the inhibition of platelet aggregation and adhesion.^20^ It has also been shown that decreased plasma eNOS levels are an important indicator of endothelial dysfunction.^9^,^21^

There are many studies indicating endothelial function is impaired in patients with SCF. Sezgin *et al.* found that endothelial function was impaired in people with SCF, and the TIMI frame count was correlated with endothelial dysfunction.^14^ Pekdemir *et al.* showed that endothelin-1 (ET-1) was higher and NO concentration was lower in patients with SCF than in a matched group of control subjects, and it was suggested that this situation was due to endothelial dysfunction.^22^ The plasma levels of NO were also found to be lower in patients with SCF than in normal subjects.^23^,^24^

In another study, plasma NO levels were found to be lower in patients with SCF than in controls, and negatively correlated with TIMI frame count.^25^ Likewise, we found in our study that plasma levels of eNOS were lower and inversely correlated with TIMI frame count in SCF patients than in control subjects. These findings support the notion that endothelial function is impaired in SCF patients.

Çamsari *et al.*. found that baseline and peak exercise ET-1 and NO concentrations were impaired in patients with SCF and suggested that endothelial dysfunction may play an active role in the pathophysiology of SCF.^26^ In our study, we found that plasma levels of eNOS involved in the synthesis of NO were significantly lower in patients with SCF than in control subjects. In addition, this decline became even more pronounced after exercise. These findings appear to support the previous studies.

Rate–pressure product (heart rate × systolic blood pressure) is well correlated with myocardial oxygen consumption. Therefore, failure of the oxygen supply to the myocardium when demand is high may result in severe cardiovascular events. In SCF patients, a negative correlation was previously reported between rate–-pressure product and post-exercise NT-proBNP levels, and a positive correlations was reported for post-exercise NO concentrations and maximal heart rate as well as exercise duration.^26^,^27^

On the basis of these concepts, the severity of ischaemia caused by exercise is generally considered to be closely related to increased ventricular wall stress or damage.^27^ In our study, we found a positive correlation between rate–pressure product and plasma levels of eNOS after exercise in SCF patients. We believe that our results are consistent with previous studies,^22^,^26^ in which lower NO levels have been found due to the response of endothelial dysfunction to increased myocardial oxygen consumption in patients with SCF.

Some limitations of this study should be considered. The design of our study was cross-sectional, and the sample size of the study population may not have been quite adequate.

## Conclusion

Our findings indicate that an important pathophysiological relationship exists between the severity of SCF in which endothelial dysfunction plays a role in the pathogenesis and level of circulating plasma levels of eNOS. To the best of our knowledge, this is the first study on this issue reported in the literature, and we believe that it will direct future large-scale studies.
